# Serum SPP1 levels are associated with disease severity and 1-year mortality in primary Sjögren’s syndrome-associated interstitial lung disease

**DOI:** 10.3389/fimmu.2026.1959993

**Published:** 2026-10-12

**Authors:** Yuting Li, Shenao Zhang, Xiaorui Ding, Qing Wang, Tong Ji, Hong Yao, Jinghong Dai

**Affiliations:** 1 Department of Pulmonary and Critical Care Medicine, Nanjing Drum Tower Hospital Clinical College of Nanjing University of Chinese Medicine, Nanjing, China; 2 Department of Pulmonary and Critical Care Medicine, The Affiliated Drum Tower Hospital of Nanjing University Medical School, Nanjing, China; 3 Department of Geriatrics, Nanjing Drum Tower Hospital Clinical College of Nanjing University of Chinese Medicine, Nanjing, China

**Keywords:** disease severity, primary Sjögren’s syndrome associated with interstitial lung disease, prognosis, secreted phosphoprotein 1, SPP1

## Abstract

Clinical significance of serum secreted phosphoprotein 1 (SPP1) in primary Sjögren’s syndrome-associated interstitial lung disease (pSS-ILD) remains unclear. In this study, we analyzed the GSE47460 lung tissue expression profile to validate SPP1 upregulation in ILD and performed GSEA to explore the potential mechanisms. A retrospective clinical cohort of 88 pSS-ILD patients and 40 healthy controls (HC) was established, with patients stratified into stable disease (SD, n=60) and acute exacerbation (AE, n=28). Serum SPP1 levels were measured by ELISA, and correlations with pulmonary function were analyzed. ROC curves, Kaplan-Meier survival analysis, and Cox regression models were used to evaluate its predictive value. SPP1 was upregulated in ILD lung tissues, with GSEA revealing enrichment of nucleotide metabolism in the high-expression group. Serum SPP1 levels were significantly elevated in pSS-ILD patients compared to HCs [37.69 (27.25–73.42) vs. 20.11 (15.68–23.72) ng/mL, P<0.001], and further increased in the AE group [85.90 (48.67–122.45) vs. 31.45 (25.87–42.02) ng/mL, P<0.001]. SPP1 levels correlated negatively with DLCO% predicted (r = −0.426, P<0.01, q = 0.013). The AUC for distinguishing AE was 0.891, and for predicting 1-year mortality was 0.712. Using a cut-off of 40.41 ng/mL, higher SPP1 levels were associated with worse survival (P = 0.002), and multivariate Cox regression identified SPP1 as an independent risk factor for 1-year mortality (HR = 1.016, 95% CI: 1.006–1.025, P = 0.001). Collectively, these clinical findings demonstrate that serum SPP1 levels correlate with disease severity and may serve as a predictor of poor prognosis in pSS-ILD.

## 1 Introduction

Primary Sjögren’s syndrome (pSS) is a chronic systemic autoimmune disease characterized by lymphocytic infiltration of the exocrine glands, manifesting clinically as xerostomia and xerophthalmia (1, 2). Beyond glandular involvement, pSS can affect multiple organ systems including the lungs, kidneys, hematopoietic system, and joints, with respiratory involvement being one of the most common extraglandular manifestations (3–5). Interstitial lung disease (ILD) represents the most frequent and severe pulmonary complication of pSS, occurring in approximately 20% of patients (6, 7). The clinical course of pSS-ILD is highly heterogeneous. While some patients may remain stable over a prolonged period, others experience progressive decline in pulmonary function or even acute exacerbation, which carries a substantially increased risk of short-term mortality (8). Current assessment of pSS-ILD relies primarily on pulmonary function tests and high-resolution computed tomography (HRCT) (9, 10). There is an urgent clinical need for simple, non-invasive, and reproducible serum biomarkers to monitor disease progression, and evaluate prognosis.

Secreted phosphoprotein 1 (SPP1), also known as osteopontin (OPN), is a multifunctional glycoprotein involved in Th1/Th17 differentiation, inflammation, tissue remodeling, and fibrosis (11, 12). SPP1 recruits immune cells, including lymphocytes, macrophages, and neutrophils, and modulates type 1 immune responses through differential regulation of IL-12 and IL-10 expression (13–15). Furthermore, SPP1 promotes fibrosis by chemoattracting macrophages and fibroblasts, modulating matrix metalloproteinases, and enhancing transforming growth factor-β (TGF-β) production (16, 17). Previous studies have demonstrated significant upregulation of SPP1 expression in lung tissues across multiple ILD subtypes, including idiopathic pulmonary fibrosis (IPF), connective tissue disease-associated ILD (CTD-ILD), pneumoconiosis, and post-COVID-19 pulmonary fibrosis (18–22), and this upregulation has been associated with disease severity and poor prognosis (23, 24). In anti-MDA5-positive dermatomyositis, SPP1 serves as a key biomarker for rapidly progressive ILD (24). Our previous multi-ILD study further confirmed SPP1 elevation in IPF and idiopathic inflammatory myopathy-associated interstitial lung disease (IIM-ILD), especially during acute exacerbation and rapid progression (25).

Despite these findings, the clinical value of SPP1 in pSS-ILD remain poorly understood. We performed transcriptomic analysis to identify ILD-associated molecular features and SPP1-related signaling pathways, and subsequently conducted a retrospective clinical study to validate the associations of serum SPP1 levels with disease activity, and prognosis in pSS-ILD patients.

## 2 Materials and methods

### 2.1 Transcriptomic analysis

Gene expression data pertaining to ILD were retrieved from the GSE47460 dataset deposited in the Gene Expression Omnibus (GEO) (26), which includes 254 patients with mixed ILD subtypes and 108 healthy lung tissue specimens. The series matrix file was obtained using the GEOquery package (27). To evaluate global transcriptional architectures, principal component analysis (PCA) was performed using FactoMineR (28), generating two-dimensional projections (PC1 vs. PC2) along with 95% confidence ellipses to delineate group segregation. Following robust quantile normalisation across arrays, differentially expressed genes (DEGs) were discerned with the limma framework (29), applying thresholds of |log_2_(fold change)| > 1 and P < 0.05 for functional interpretation. DEGs were subjected to Kyoto Encyclopedia of Genes and Genomes (KEGG) pathway enrichment (30), and gene set enrichment analysis (GSEA), both executed through the clusterProfiler package (31), with statistical significance defined by a false discovery rate (FDR) below 0.05. In addition, SPP1 co-expression analysis was performed using the same dataset. The top 50 SPP1-co-expressed genes were used to construct a co-expression network, and Gene Ontology (GO) enrichment analysis of these genes was conducted using the clusterProfiler package. Furthermore, the expression dynamics of the pivotal gene SPP1 were extracted from the normalised matrix, and intergroup comparisons were carried out employing the Wilcoxon rank-sum test, with p < 0.05 deemed significant, and the results rendered as violin plots (32).

### 2.2 Study population

This study retrospectively enrolled 88 patients diagnosed with pSS-ILD at Nanjing Drum Tower Hospital between July 2018 and July 2024, and recruited 40 age- and sex-matched healthy controls (HC) from the Health Examination Center of our hospital (**Figure 1**). The inclusion criteria were as follows: 1) diagnosis of primary Sjögren’s syndrome according to the 2016 ACR/EULAR classification criteria (33); 2) ILD confirmed by HRCT and reviewed independently by two experienced radiologists and one pulmonologist; 3) age ≥ 18 years. The exclusion criteria were: 1) combined other connective tissue diseases; 2) malignancies; 3) acute infections. HRCT images were independently reviewed by two experienced chest radiologists and classified as NSIP, OP, UIP, or LIP patterns according to the predominant pattern based on the 2013 American Thoracic Society classification of idiopathic interstitial pneumonia guidelines (34). Any disagreements were resolved by consultation.

**Figure 1:**
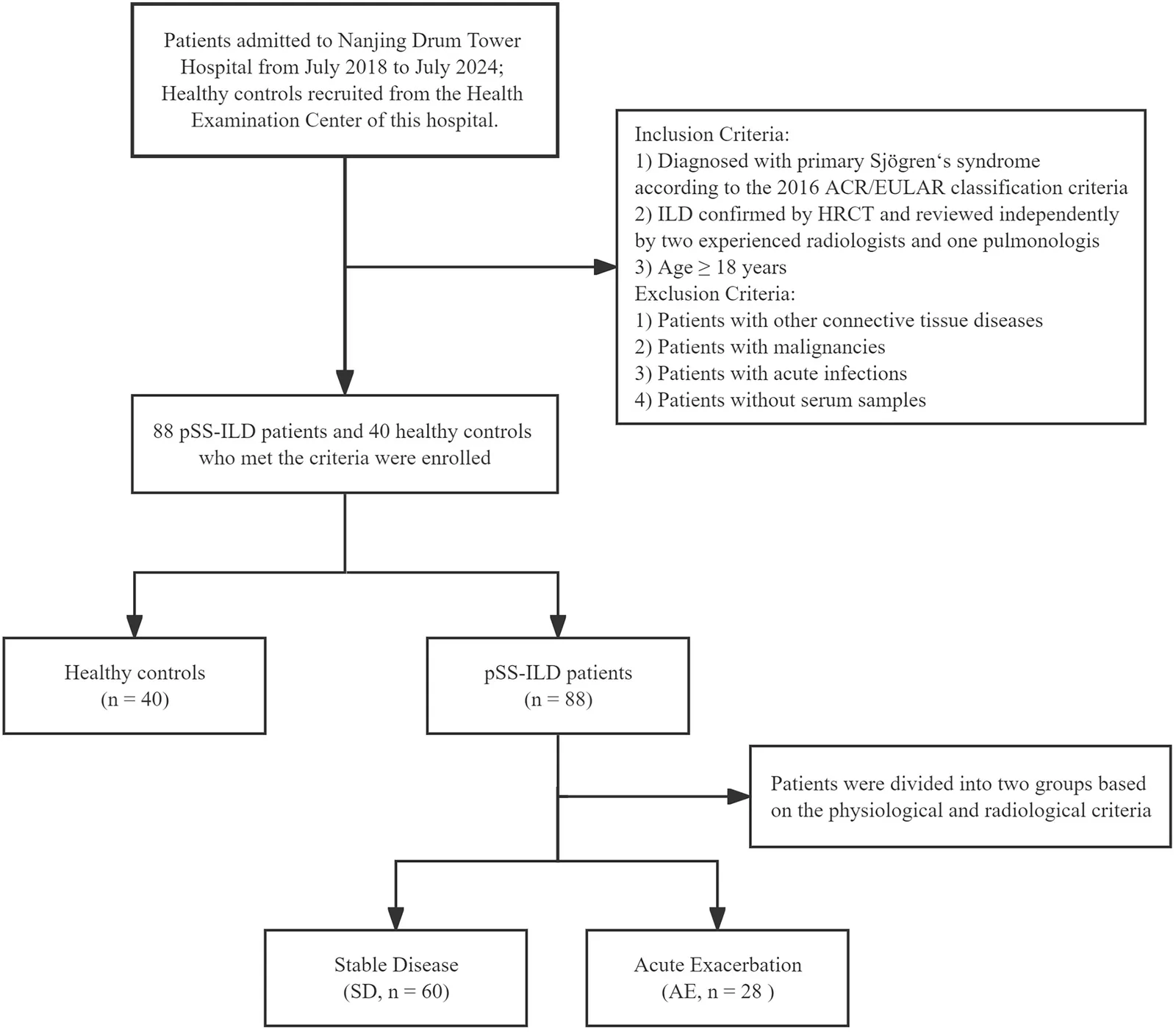
Study flow chart. A total of 88 patients with pSS-ILD and 40 healthy controls were enrolled. Patients were divided into stable disease (SD, n=60) and acute exacerbation (AE, n=28) groups based on the inclusion and exclusion criteria. pSS-ILD, primary Sjögren’s syndrome-associated interstitial lung disease.

Based on clinical manifestations and disease course, patients with pSS-ILD were divided into two groups: stable disease (SD, n=60) and acute exacerbation (AE, n=28). Currently, there is no universally accepted international consensus definition for acute exacerbation in pSS-ILD. In clinical practice and research, the diagnosis of AE in pSS-ILD is generally extrapolated from the diagnostic framework for acute exacerbation of idiopathic pulmonary fibrosis (AE-IPF). Within this framework, AE in pSS-ILD was defined as an acute, clinically significant respiratory deterioration occurring within a short period (typically less than one month), accompanied by new bilateral ground-glass opacities and/or consolidations on HRCT superimposed on a background of pre-existing ILD, and not fully explained by cardiac failure, fluid overload, or other identifiable causes such as infection or pulmonary embolism (35–37).

### 2.3 Data collection

Demographic data, baseline pulmonary function test results, laboratory findings, and treatment information were retrospectively collected through review of the electronic medical record system. Baseline pulmonary function tests (PFTs) were performed at diagnosis included forced vital capacity (FVC), forced expiratory volume in one second (FEV1), and diffusing capacity of the lung for carbon monoxide (DLCO). Laboratory parameters included oxygenation index (PaO_2_/FiO_2_), routine hematological indices, inflammatory markers such as C-reactive protein (CRP), serum biochemical parameters including albumin (Alb) and lactate dehydrogenase (LDH), coagulation markers like D-dimer, and immunological parameters including immunoglobulins and complement levels. Autoantibody profiles, including antinuclear antibody (ANA) and anti-Ro52 antibody, were also collected. Treatment information documented medication regimens at baseline and during follow-up, covering glucocorticoids, immunosuppressive agents, and antifibrotic agents. The requirement for written informed consent was waived by the Ethics Committee for all patients, including deceased patients. This study was approved by the Ethics Committee of Nanjing Drum Tower Hospital (Approval No.: 2025-0795-02).

### 2.4 Measurement of serum SPP1 levels by ELISA

Fasting venous blood samples were collected from all participants at diagnosis and immediately stored at −80 °C until analysis. Serum SPP1 concentrations were quantitatively determined using a commercially available human SPP1 enzyme-linked immunosorbent assay (ELISA) kit (Elabscience, China) according to the manufacturer’s instructions. Briefly, frozen serum samples were thawed at 4 °C and centrifuged briefly, and the supernatants were used for detection. Standards and diluted serum samples were added to microplates pre-coated with capture antibodies. After incubation and washing, biotin-labeled detection antibodies and horseradish peroxidase-labeled avidin were added sequentially. Finally, chromogenic substrate solution was added for color development in the dark, and the reaction was terminated with stop solution. The optical density of each well was immediately measured at 450 nm using a microplate reader, and SPP1 concentrations (ng/mL) were calculated based on the standard curve.

### 2.5 Follow-up and outcomes

All enrolled patients were followed up for at least 12 months after enrollment. Follow-up was conducted through regular outpatient visits and telephone interviews. The primary endpoint was all-cause mortality within one year from baseline. Survival data were obtained from the electronic medical record system and confirmed by telephone follow-up. For deceased patients, the date and cause of death were recorded.

### 2.6 Statistical analysis

All statistical analyses were performed using R software (version 4.3.2, R Core Team, Vienna, Austria), SPSS (version 27.0, IBM Corp., Armonk, NY, USA), and GraphPad Prism (version 10, GraphPad Software, San Diego, CA, USA). All tests were two-sided, and a P value < 0.05 was considered statistically significant.

Normality of all continuous variables was assessed using the Shapiro-Wilk test. For multiple comparisons, the Benjamini-Hochberg false discovery rate (BH-FDR) method was used, and both unadjusted and FDR-adjusted P values (q values) were reported. Missing data were handled by complete-case analysis. Continuous variables with normal distribution are presented as mean ± standard deviation (SD) and compared using the independent samples t-test. Non-normally distributed variables are expressed as median (interquartile range, IQR) and compared using the Mann-Whitney U test. Categorical variables are presented as frequencies (percentages) and analyzed using the chi-square test or Fisher’s exact test.

Correlations between SPP1 levels and pulmonary function parameters, as well as PaO_2_/FiO_2_, D-dimer, CRP, and LDH, were assessed using Spearman’s rank correlation coefficient. Receiver operating characteristic (ROC) curves were constructed to determine the optimal cut-off value of SPP1 for predicting AE and 1-year mortality, using the Youden index. Internal validation was performed using 1,000 bootstrap resamples with replacement to estimate optimism-corrected AUCs and cut-off stability. The Kaplan-Meier method with the log-rank test was used to compare survival between groups stratified by the SPP1 cut-off. Univariate and multivariate Cox proportional hazards regression models were employed to identify predictors of disease progression and all-cause mortality, with results reported as hazard ratios (HR) and 95% confidence intervals (CI). To further assess the robustness of the independent prognostic value of SPP1, sensitivity analyses were performed. A Firth-penalized Cox regression was also performed to correct for potential estimation bias due to the limited number of events (n = 14).

## 3 Results

### 3.1 Transcriptomic analysis identifies SPP1 as a key upregulated gene in ILD

Differential expression analysis of GSE47460 (254 ILD, 108 controls) was performed to identify ILD-associated molecular features. Principal component analysis showed that ILD patients and healthy controls were largely separated by their 95% confidence ellipses, suggesting significantly different transcriptomic profiles between the two groups (**Figure 2A**). Using thresholds of |log_2_FC| > 1 and adj.P < 0.05, 163 DEGs were identified, including 93 upregulated and 70 downregulated genes. Notably, SPP1 was among the most significantly upregulated genes (log_2_FC = 1.931, adj.P = 9.76×10^-^¹¹) (**Figure 2B**). Heatmap analysis showed that the top 50 DEGs clearly distinguished ILD patients from healthy controls, with SPP1 displaying a consistent high-expression pattern across the ILD group (**Figure 2C**).

**Figure 2:**
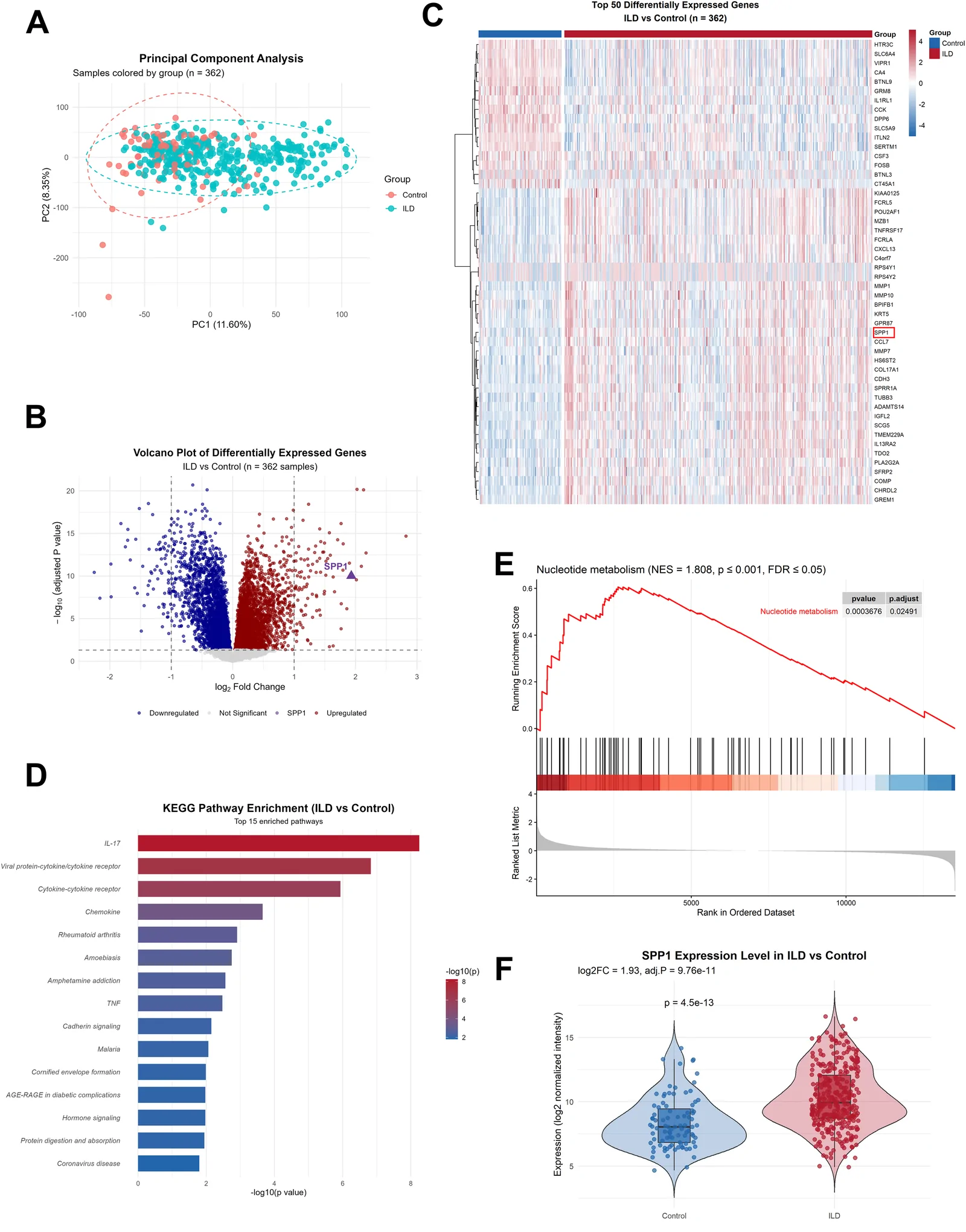
Transcriptomic profiling of ILD versus healthy controls from the GSE47460 dataset. **(A)** Principal component analysis (PCA) plot showing the distribution of 254 ILD patients and 108 healthy controls. The first principal component (PC1) and second principal component (PC2) explained 11.60% and 8.35% of the variance, respectively. **(B)** Volcano plot of differentially expressed genes between ILD patients and healthy controls. Red dots represent significantly upregulated genes (log_2_FC > 1, adj.P < 0.05), blue dots represent significantly downregulated genes (log_2_FC < −1, adj.P < 0.05), and gray dots represent genes with no significant difference. SPP1 is highlighted as one of the most significantly upregulated genes. **(C)** Heatmap of the top 50 differentially expressed genes, showing clear separation between ILD patients and healthy controls. Red represents high expression and blue represents low expression. SPP1 displays a bright red high-expression band consistently across the ILD group. **(D)** KEGG pathway enrichment analysis of differentially expressed genes showing significant enrichment of IL-17 signaling pathway, cytokine-cytokine receptor interaction, chemokine signaling pathway, and TNF signaling pathway. **(E)** Gene set enrichment analysis (GSEA) plot showing that nucleotide metabolism (NES = 1.808, p < 0.001, FDR q ≤ 0.05) was the most significantly upregulated pathway in the SPP1 high-expression group, with its enrichment curve showing a pronounced peak at the front of the ranked list. **(F)** Boxplot showing SPP1 expression levels in ILD patients and healthy controls. SPP1 expression in the ILD group was significantly higher than that in healthy controls (log_2_FC = 1.931, adj.P = 9.76×10^-^¹¹). ILD, interstitial lung disease; SPP1, secreted phosphoprotein 1; KEGG, Kyoto Encyclopedia of Genes and Genomes; NES, normalized enrichment score; FC, fold change; adj.P, adjusted P value.

KEGG pathway enrichment analysis revealed significant enrichment of the IL-17 signaling pathway, cytokine-cytokine receptor interaction, chemokine signaling pathway, and TNF signaling pathway (**Figure 2D**). To explore SPP1’s function, samples were stratified by median SPP1 expression for GSEA. Among 18 significant KEGG pathways (q < 0.05), 6 were upregulated and 12 downregulated in the high-SPP1 group. The top upregulated pathways were nucleotide metabolism (NES = 1.808, p < 0.001, FDR q ≤ 0.05) (**Figure 2E**), ribosome (NES = 1.724, q = 0.004), p53 signaling (NES = 1.702, q = 0.021), and cell cycle (NES = 1.556, q = 0.026). The most downregulated pathways were NK cell-mediated cytotoxicity (NES = −1.990, q < 0.001) and MAPK signaling (NES = −1.451, q = 0.024). The SPP1 co-expression network (**Supplementary Figure S1A**) showed that SPP1 was positively correlated with MMP1, LAMA3 and CCL7, etc., and negatively correlated with CAV1, CAV2, WNT7A, etc. GO enrichment analysis showed that SPP1 co-expressed genes were mainly involved in cell junction assembly, cell migration, epithelial cell proliferation, cell adhesion, extracellular matrix organization, and angiogenesis (**Supplementary Figure S1B**).

Boxplot analysis confirmed significantly higher SPP1 expression in the ILD group than in healthy controls (log_2_FC = 1.931, adj.P = 9.76×10^-^¹¹) (**Figure 2F**), consistent with the volcano plot and heatmap findings.

### 3.2 Baseline characteristics of the study population

A total of 88 patients with pSS-ILD and 40 HCs were enrolled in this study. As shown in **Table 1**, there were no significant differences between the pSS-ILD and HC groups in age (62.44 ± 9.92 vs. 63.98 ± 7.22 years, P = 0.327) or sex distribution (male: 22.7% vs. 37.5%, P = 0.082), confirming the comparability of the two cohorts. The distribution of HRCT patterns is shown in **Supplementary Table S1**. NSIP was the predominant pattern (70.5%), followed by UIP (23.9%), LIP (4.5%), and OP (1.1%). The distribution did not differ significantly between SD and AE groups (NSIP, P = 0.386; UIP, P = 0.213; LIP, P = 0.764; OP, P = 0.492).

**Table 1:** Characteristics in HCs and pSS-ILD patients.

Characteristics	HC (n=40)	pSS-ILD (n=88)	P
Age, years	63.98 ± 7.22	62.44 ± 9.92	0.327
Sex, M(%)	15.0 (37.5%)	20.0 (22.7%)	0.082
SPP1, ng/mL	20.11 (15.68-23.72)	37.69 (27.25-73.42)	<0.001

Data were expressed as mean ± standard deviation, median (IQR), or n (%), where appropriate. HC, healthy control; pSS-ILD, primary Sjögren's syndrome-associated interstitial lung disease; SPP1, secreted phosphoprotein 1.

Baseline clinical characteristics were compared between SD (n=60) and AE (n=28) groups (**Table 2**). In terms of demographics, the two groups showed no significant differences in age (P = 0.306), sex (P = 0.371), smoking history (P = 0.720), or body mass index (P = 0.221). Regarding respiratory function, the AE group had a significantly lower PaO_2_/FiO_2_ ratio compared to the SD group (321.61 ± 94.16 vs. 381.34 ± 91.28 mmHg, P = 0.006). Pulmonary function tests revealed that AE patients had significantly lower absolute DLCO (2.21 ± 1.22 vs. 3.36 ± 1.40, P = 0.023) and DLCO% predicted [20.15 (16.50–33.40)% vs. 45.60 (35.30–61.80)%, P<0.001] than SD patients, as well as lower absolute FVC (1.46 ± 0.54 vs. 1.87 ± 0.63, P = 0.016). Laboratory findings showed that AE patients had significantly higher levels of D-dimer [0.98 (0.38–2.79) vs. 0.35 (0.21–0.76) mg/L, P<0.001], lactate dehydrogenase (LDH) [264.00 (239.00–345.00) vs. 232.00 (194.50–269.25) U/L, P<0.001], and C-reactive protein (CRP) [9.70 (5.90–49.65) vs. 4.50 (2.85–8.68) mg/L, P<0.001], while serum albumin levels were significantly lower in the AE group (35.11 ± 4.37 vs. 37.15 ± 3.73 g/L, P = 0.026). No significant differences were observed in autoantibody positivity rates between the two groups. In terms of treatment, the use of glucocorticoids and antifibrotic agents was comparable between groups, but immunosuppressant use was significantly more common in the SD group than in the AE group (73.3% vs. 50.0%, P = 0.031). During follow-up, the 1-year mortality rate was significantly higher in the AE group than in the SD group (39.3% vs. 5.0%, P<0.001).

**Table 2:** Baseline clinical characteristics in pSS-ILD patients.

Characteristics	SD (n=60)	AE (n=28)	P
Demographic			
Age, years	61.70 ± 9.90	64.04 ± 9.95	0.306
Sex, M (%)	12 (20.0%)	8 (28.6%)	0.371
Smoking history, n (%)	6 (10.0%)	4 (14.3%)	0.720
BMI, Kg/m2	23.19 ± 3.09	24.19 ± 3.80	0.221
Comorbidity			
Pulmonary hypertension	16 (26.7%)	8 (28.6%)	0.852
Diabetes	11 (18.3%)	3 (10.7%)	0.534
Hypertension	17 (28.3%)	5 (17.9%)	0.290
Pulmonary function testing			
FVC	1.87 ± 0.63 (n=43)	1.46 ± 0.54 (n=16)	0.016
FVC% predicted	70.19 ± 18.05 (n=43)	61.94 ± 14.74 (n=15)	0.117
FEV1	1.55 ± 0.51 (n=42)	1.31 ± 0.45 (n=16)	0.083
FEV1% predicted	70.82 ± 19.23 (n=42)	64.92 ± 16.75 (n=15)	0.297
DLCO	3.36 ± 1.40 (n=35)	2.21 ± 1.22 (n=10)	0.023
DLCO% predicted	45.60 (35.30-61.80) (n=35)	20.15 (16.50-33.40) (n=10)	<0.001
Laboratory examination			
PO2, mmHg	85.00 (74.50-96.75)	84.50 (68.75-102.00)	0.635
PO2/FiO2, mmHg	381.34 ± 91.28	321.61 ± 94.16	0.006
D-dimer,mg/L	0.35 (0.21-0.76)	0.98 (0.38-2.79)	<0.001
WBC, 109/L	6.60 (5.25-9.08)	7.75 (5.68-10.05)	0.278
RBC, 1012/L	4.13 (3.94-4.47)	4.20 (3.94-4.48)	0.943
LYM%, %	23.11 ± 10.36	21.56 ± 14.15	0.565
LYM,109/L	1.45 (1.12-1.80)	1.40 (0.80-2.10)	0.495
N%, %	66.68 ± 11.73	69.60 ± 17.94	0.437
N,109/L	4.45 (2.98-6.48)	5.65 (3.08-7.98)	0.309
Hb, g/L	124.67 ± 15.03	124.21 ± 17.39	0.901
PLT, 109/L	211.37 ± 75.42	208.57 ± 91.54	0.880
Alb, g/L	37.15 ± 3.73	35.11 ± 4.37	0.026
TCHO, mmol/L	4.27 (3.59-5.12)	4.46 (3.79-5.41)	0.504
TG, mmol/L	1.35 (0.94-1.61)	1.19 (0.84-1.77)	0.764
ALT, U/L	18.7 (8.83-22.45)	19.35 (9.10-38.18)	0.299
AST, U/L	17.75 (13.90-22.65)	21.20 (15.83-28.28)	0.067
CREA, umol/L	51.50 (44.00-61.75)	52.00 (47.00-61.75)	0.603
BUN, mmol/L	5.05 (3.80-6.08)	4.70 (3.83-6.75)	0.939
GLU, mmol/L	4.65 (4.27-5.52)	4.71 (4.16-5.97)	0.632
GLB, g/L	26.60 (23.58-31.45)	28.60 (25.10-34.78)	0.065
LDH, U/L	232.00 (194.50-269.25)	264.00 (239.00-345.00)	<0.001
TP, g/L	63.95 (60.30-69.75)	65.45 (60.63-76.55)	0.690
CRP, mg/L	4.50 (2.85-8.68)	9.70 (5.90-49.65)	<0.001
HDL, mmol/L	1.97 (1.07-1.85)	1.34 (0.99-2.43)	0.993
LDL, mmol/L	2.05 (1.58-3.05)	2.04 (1.19-2.97)	0.465
CK, U/L	46.00 (33.00-80.00)	47.50 (31.50-79.75)	0.805
CK-MB, U/L	14.00 (12.00-16.00)	14.00 (9.25-15.75)	0.237
C3, g/L	1.12 (1.01-1.23)	1.22 (1.08-1.29)	0.027
C4, g/L	0.22 ± 0.08	0.25 ± 0.12	0.261
IgA, g/L	2.19 (1.68-3.09)	2.91 (1.92-3.75)	0.114
IgE, mg/L	32.50 (11.50-92.50)	43.50 (19.75-250.25)	0.311
IgG, g/L	12.15 (9.50-14.88)	13.65 (9.93-17.84)	0.261
IgM, g/L	1.04 (0.77-1.48)	1.33 (0.89-1.61)	0.139
Antibody			
ANA	46 (76.7%)	21 (75.0%)	1.000
Ro52	39 (65.0%)	18 (64.3%)	1.000
Therapy during follow-up			
Glucocorticoid therapy	51 (85.0%)	20 (71.4%)	0.154
Antifibrotic treatment	11 (18.3%)	3 (10.7%)	0.534
Immunosuppressant	44 (73.3%)	14 (50.0%)	0.031
One year mortality	3 (5.0%)	11 (39.3%)	<0.001
SPP1, ng/mL	31.45 (25.87-42.02)	85.90 (48.67-122.45)	<0.001

Data are expressed as mean ± standard deviation, median (IQR), or n (%), where appropriate. SD, stable disease; AE, acute exacerbation; M, males; BMI, body mass index; FVC, forced vital capacity; FEV1, forced expiratory volume in 1 second; DLCO, diffusing capacity of the lung for carbon monoxide; PO_2_, partial pressure of oxygen; PaO_2_/FiO_2_, ratio of arterial oxygen partial pressure to fractional inspired oxygen; WBC, white blood cell; RBC, red blood cell; LYM, lymphocyte; N, neutrophil; Hb, hemoglobin; PLT, platelet; Alb, albumin; TCHO, total cholesterol; TG, triglycerides; ALT, alanine aminotransferase; AST, aspartate aminotransferase; CREA, creatinine; BUN, blood urea nitrogen; GLU, glucose; GLB, globulin; LDH, lactate dehydrogenase; TP, total protein; CRP, C-reactive protein; HDL, high-density lipoprotein; LDL, low-density lipoprotein; CK, creatine kinase; CK-MB, creatine kinase-MB; C3, complement component 3; C4, complement component 4; IgA, immunoglobulin A; IgE, immunoglobulin E; IgG, immunoglobulin G; IgM, immunoglobulin M; ANA, antinuclear antibody; Ro52, anti-Ro52 antibody; SPP1, secreted phosphoprotein 1.

### 3.3 Correlation of serum SPP1 with pulmonary function and other laboratory and physiological parameters

Spearman correlation analysis was performed to evaluate the relationship between serum SPP1 levels and pulmonary function parameters in pSS-ILD patients (**Table 3**). Serum SPP1 levels were significantly negatively correlated with DLCO% predicted (r = -0.426, P < 0.01, q = 0.013). A negative correlation trend was also observed between SPP1 and absolute DLCO, although this did not reach statistical significance (r = -0.264, P = 0.093, q = 0.133). However, no significant correlations were observed between SPP1 and FVC (r = -0.085, P = 0.581, q = 0.875), FVC% predicted (r = 0.035, P = 0.822, q = 0.886), FEV1 (r = -0.054, P = 0.731, q = 0.886), or FEV1% predicted (r = 0.010, P = 0.948, q = 0.940). In the overall cohort, serum SPP1 was significantly positively correlated with D-dimer (r = 0.378, P < 0.001, q = 0.005) and CRP (r = 0.282, P = 0.011, q = 0.039), whereas correlations with LDH (r = 0.236, P = 0.031, q = 0.085) and PaO_2_/FiO_2_ (r = −0.195, P = 0.069, q = 0.125) did not remain significant after FDR correction (**Supplementary Figure S2**).

**Table 3:** Correlation analysis.

Variable	SPP1	FVC	FVC%	FEV1	FEV1%	DLCO	DLCO%
SPP1	1						
FVC	-0.085	1					
FVC%	0.035	0.749**	1				
FEV1	-0.054	0.908**	0.753**	1			
FEV1%	0.010	0.720**	0.926**	0.823**	1		
DLCO	-0.264	0.822**	0.524**	0.783**	0.551**	1	
DLCO%	-0.426**	0.682**	0.552**	0.627**	0.550**	0.776**	1

Data are presented as Spearman‘s correlation coefficients (r). **Correlation is significant at the 0.01 level (2-tailed);. SPP1, secreted phosphoprotein 1; FVC, forced vital capacity; FEV1, forced expiratory volume in 1 second; DLCO, diffusing capacity of the lung for carbon monoxide.

### 3.4 Serum SPP1 levels in pSS-ILD patients

Serum SPP1 concentrations were measured by ELISA in all participants. The median serum SPP1 level in the HC group (n=40) was 20.11 ng/mL (IQR: 15.68–23.72), whereas that in the pSS-ILD group (n=88) was 37.69 ng/mL (IQR: 27.25–73.42), with a statistically significant difference between the two groups (P<0.001) (**Figure 3A**). Further subgroup analysis revealed that serum SPP1 levels in the AE group were significantly higher than those in the SD group [85.90 (48.67–122.45) ng/mL vs. 31.45 (25.87–42.02) ng/mL, P<0.001] (**Figure 3B**). Based on survival status during the 1-year follow-up period, pSS-ILD patients were divided into survivors (n=74) and non-survivors (n=14). The median serum SPP1 level in survivors was 34.18 ng/mL (IQR: 27.09–58.43), whereas that in non-survivors was significantly higher at 65.83 ng/mL (IQR: 42.30–105.99), with a statistically significant difference between the two groups (P = 0.012) (**Figure 3C**).

**Figure 3:**
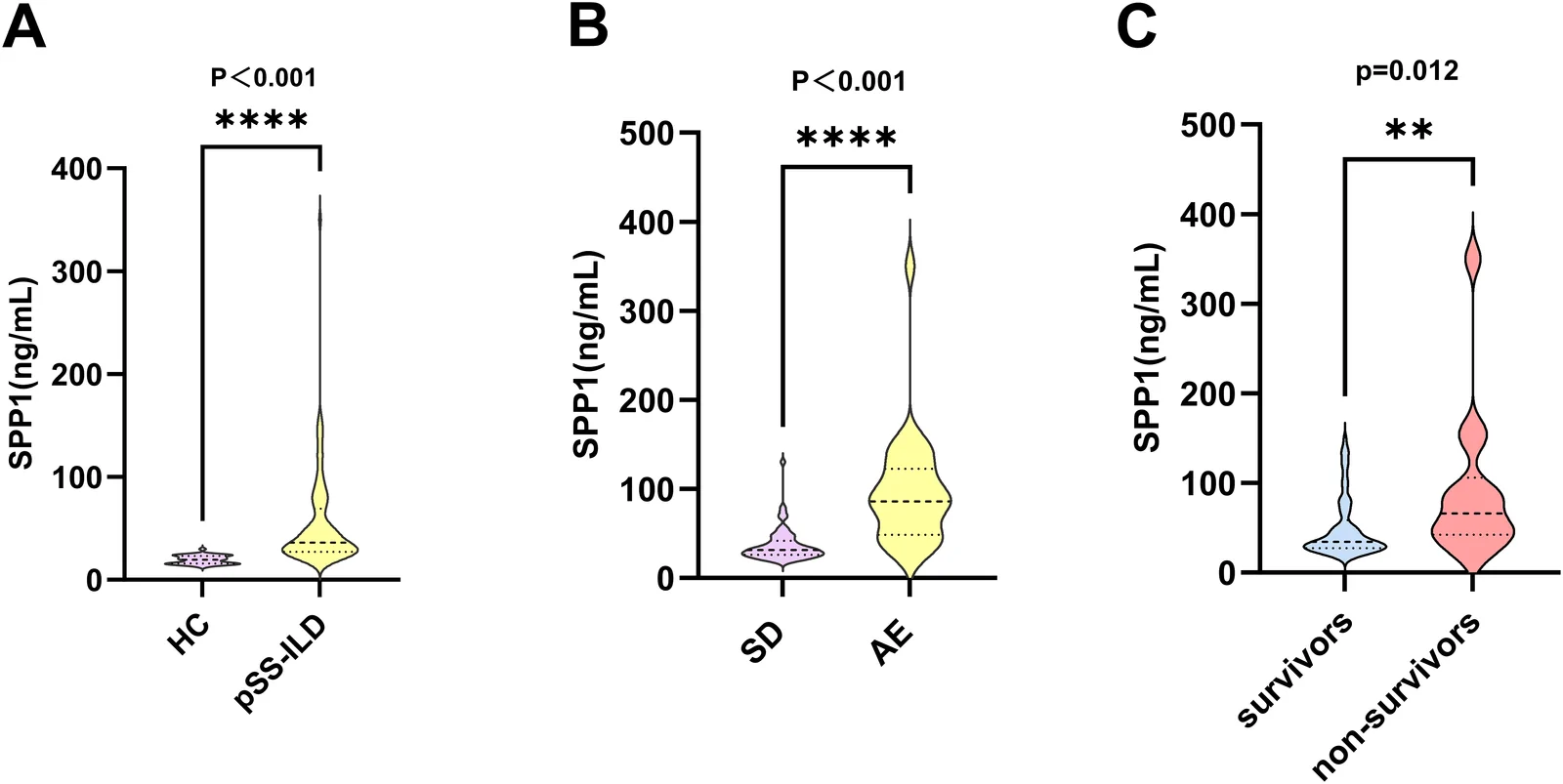
Serum SPP1 levels. **(A)** Serum SPP1 levels in healthy controls (HC, n=40) and pSS-ILD patients (n=88). Serum SPP1 levels were significantly elevated in pSS-ILD patients compared to healthy controls [37.69 (27.25–73.42) vs. 20.11 (15.68–23.72) ng/mL, P < 0.001]. **(B)** Serum SPP1 levels in stable disease (SD, n=60) and acute exacerbation (AE, n=28) groups. Serum SPP1 levels were markedly higher in the AE group than in the SD group [85.90 (48.67–122.45) vs. 31.45 (25.87–42.02) ng/mL, P < 0.001]. **(C)** Serum SPP1 levels in survivors (n=74) and non-survivors (n=14). Non-survivors had significantly higher serum SPP1 levels than survivors [65.83 (42.30–105.99) vs. 34.18 (27.09–58.43) ng/mL, P = 0.012]. Data are presented as median with interquartile range. ****P < 0.001, **P < 0.05. SPP1, secreted phosphoprotein 1; HC, healthy controls; pSS-ILD, primary Sjögren’s syndrome-associated interstitial lung disease; SD, stable disease; AE, acute exacerbation.

### 3.5 SPP1 is associated with disease progression in pSS-ILD

To further evaluate the clinical utility of serum SPP1 in identifying acute exacerbation, we constructed ROC curves (**Figure 4A**). The area under the curve (AUC) for distinguishing AE from SD was 0.891 (95% CI: 0.827–0.955, P < 0.001), indicating excellent discriminative performance. The optimal cut-off value determined by the Youden index was 47.49 ng/mL, yielding a sensitivity of 82.1% and a specificity of 83.3%. Bootstrap internal validation showed an optimism-corrected AUC of 0.891 (95% CI: 0.800–0.964) for AE, with a bootstrap median cut-off of 47.50 ng/mL (**Supplementary Table S2**).

**Figure 4:**
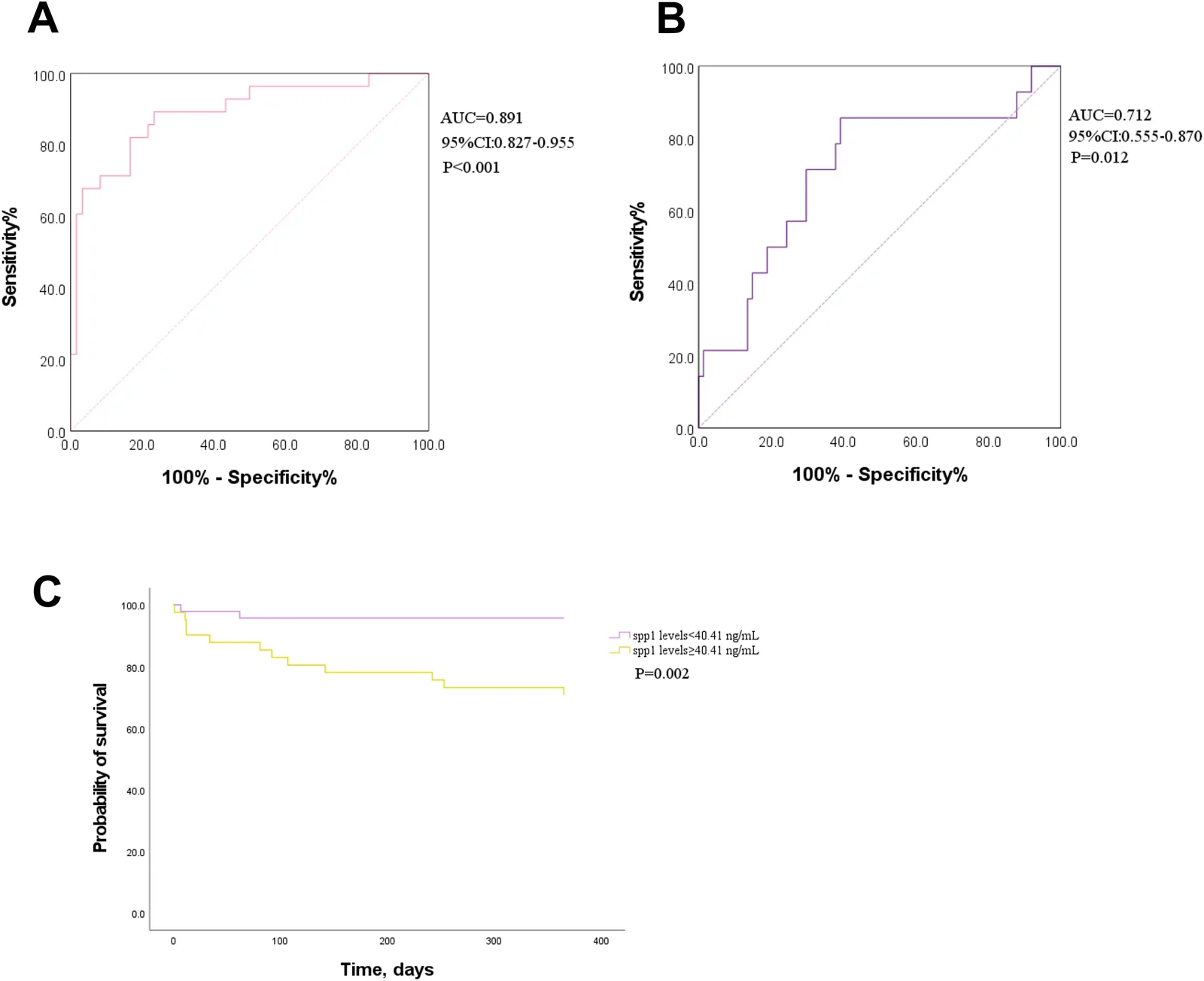
Prognostic value of serum SPP1 in pSS-ILD patients. **(A)** ROC curve of serum SPP1 for distinguishing acute exacerbation (AE) from stable disease (SD). The AUC was 0.891 (95% CI: 0.827–0.955, P < 0.001). The optimal cut-off value determined by the Youden index was 47.49 ng/mL, yielding a sensitivity of 82.1% and a specificity of 83.3%. **(B)** ROC curve of serum SPP1 for predicting one-year mortality. The AUC was 0.712 (95% CI: 0.555–0.870, P = 0.012). The optimal cut-off value was 40.41 ng/mL, corresponding to a sensitivity of 85.7% and a specificity of 60.8%. **(C)** Kaplan-Meier survival curves for pSS-ILD patients stratified by serum SPP1 levels using the optimal cut-off value of 40.41 ng/mL. Patients with SPP1 ≥ 40.41 ng/mL had significantly lower one-year cumulative survival rates than those with SPP1 < 40.41 ng/mL (log-rank: P = 0.002). SPP1, secreted phosphoprotein 1; pSS-ILD, primary Sjögren’s syndrome-associated interstitial lung disease; ROC, receiver operating characteristic; AUC, area under the curve; CI, confidence interval.

### 3.6 Prognostic value of SPP1 for 1-year mortality in pSS-ILD

We assessed the predictive value of SPP1 for 1-year mortality using ROC curve analysis (**Figure 4B**), with an AUC of 0.712 (95% CI: 0.555–0.870, P = 0.012). The optimal cut-off value derived from the Youden index was 40.41 ng/mL, corresponding to a sensitivity of 85.7% and a specificity of 60.8%. Bootstrap internal validation yielded an optimism-corrected AUC of 0.710 (95% CI: 0.537–0.860) for 1-year mortality, with a bootstrap median cut-off of 40.40 ng/mL (**Supplementary Table S2**). Based on this cut-off value, patients were stratified into high SPP1 (≥ 40.41 ng/mL) and low SPP1 (< 40.41 ng/mL) groups. Kaplan-Meier survival analysis (**Figure 4C**) revealed that patients in the high SPP1 group had a significantly lower 1-year cumulative survival rate compared to those in the low SPP1 group (P = 0.002).

Univariate and multivariate Cox proportional hazards regression models were employed to further validate the independent prognostic value of serum SPP1 for 1-year mortality in pSS-ILD patients (**Table 4**). Univariate Cox regression analysis identified multiple risk factors for 1-year mortality, including older age (P = 0.032), lower oxygenation index (P < 0.001), higher D-dimer (P = 0.039), higher white blood cell count (P = 0.010), higher neutrophil count (P = 0.002), higher lactate dehydrogenase (P < 0.001), higher C-reactive protein (P = 0.002), and higher SPP1 levels (P < 0.001). After BH-FDR correction for multiple comparisons, oxygenation index (q = 0.002), white blood cell count (q = 0.047), neutrophil count (q = 0.013), lactate dehydrogenase (q = 0.002), C-reactive protein (q = 0.013), and SPP1 (q = 0.003) remained statistically significant, whereas age (q = 0.100) and D-dimer (q = 0.115) did not (**Supplementary Table S3**). Multivariate Cox regression analysis, after adjusting for age and sex, demonstrated that serum SPP1 remained an independent risk factor for 1-year mortality (HR = 1.016, 95% CI: 1.006–1.025, P = 0.001). Sensitivity analyses further confirmed the robustness of this finding (**Supplementary Table S4**). Across all models additionally adjusted for PaO_2_/FiO_2_, D-dimer, BUN, GLU, or CRP, the HR of SPP1 remained consistent in direction and magnitude (HR 1.011–1.016) and statistically significant (P = 0.001–0.044). The Firth-penalized Cox regression also yielded a consistent result (HR = 1.015, 95% CI: 1.006–1.025, P = 0.002).

**Table 4:** The Cox proportional hazard model for prognostic factors of pSS-ILD patients.

Variable	Univariate		Multivariate	
	HR (95% CI)	P	HR (95% CI)	P
Age, years	1.065 (1.005-1.127)	0.032	1.059 (0.993-1.129)	0.080
Sex, M (%)	0.924 (0.258-3.314)	0.904	1.792 (0.489-6.564)	0.378
Smoking history, n (%)	0.778 (0.174-3.475)	0.742		
PO_2_/FiO_2_, mmHg	0.988 (0.982-0.994)	<0.001		
D-dimer,mg/L	1.057 (1.003-1.114)	0.039		
WBC, 10^9^/L	1.215 (1.047-1.410)	0.010		
N,10^9^/L	1.239 (1.080-1.415)	0.002		
ALT, U/L	1.014 (1.002-1.026)	0.026		
BUN, mmol/L	1.162 (1.041-1.297)	0.007		
GLU, mmol/L	1.562 (1.307-1.867)	<0.001		
LDH, U/L	1.004 (1.002-1.005)	<0.001		
CRP, mg/L	1.023 (1.009-1.038)	0.002		
CK-MB, U/L	1.087 (1.012-1.167)	0.022		
SPP1, ng/mL	1.017 (1.008-1.027)	<0.001	1.016 (1.006-1.025)	0.001

HR, hazard ratio; CI, confidence interval; M, male; PaO_2_/FiO_2_, ratio of arterial oxygen partial pressure to fractional inspired oxygen; WBC, white blood cell count; N, neutrophil; ALT, alanine aminotransferase; BUN, blood urea nitrogen; GLU, glucose; LDH, lactate dehydrogenase; CRP, C-reactive protein; CK-MB, creatine kinase-MB; SPP1, secreted phosphoprotein 1. Age, Sex and SPP1 were included in the multivariate model.

## 4 Discussion

Transcriptomic analysis of a mixed ILD cohort suggested SPP1 upregulation in ILD lung tissues, providing a rationale for subsequent validation in our pSS-ILD clinical cohort. Serum SPP1 was significantly higher in pSS-ILD patients than in HCs, with further elevation in AE and non-survivors. SPP1 showed excellent discriminative ability for acute exacerbation (AUC = 0.891) and good predictive value for 1-year mortality (AUC = 0.712), and remained an independent predictor after multivariate adjustment (HR = 1.016, P = 0.001). These findings support serum SPP1 as a potential biomarker for disease monitoring, risk stratification, and prognostic assessment in pSS-ILD.

Transcriptomic analysis revealed the potential functional roles of SPP1 in ILD at the molecular level. KEGG analysis showed that DEGs were significantly enriched in the IL-17, chemokine, and TNF signaling pathways, all closely associated with inflammation and immune regulation. Previous studies have shown that SPP1 promotes Th17 responses via Irf7 inhibition (38), and drives M2 macrophage polarization (39), suggesting that elevated SPP1 may contribute to a pulmonary inflammatory microenvironment. GSEA further showed upregulation of cell proliferation-related pathways in the SPP1 high-expression group, including nucleotide metabolism (NES = 1.808), ribosome (NES = 1.724), p53 signaling (NES = 1.702), and cell cycle (NES = 1.556). SPP1 has been shown to be expressed in multiple pro-fibrotic cell types, including macrophages, epithelial cells, and fibroblasts, and promotes fibrosis via JAK2/STAT3 (39) and PI3K/Akt/mTOR signaling (40). These findings suggest that high SPP1 expression may support cell proliferation through nucleotide and protein synthesis, contributing to ILD fibrosis. Meanwhile, NK cell-mediated cytotoxicity (NES = -1.990) and MAPK signaling (NES = -1.451) were downregulated, consistent with reports that SPP1 impairs anti-tumor immunity by limiting T/NK cell infiltration via the SPP1-CD44 axis (41, 42). Notably, although the GSE47460 dataset includes mixed ILD subtypes and cannot be used to isolate pSS-ILD samples, the consistent upregulation of SPP1 across subtypes supports its potential as a common molecular marker for ILD, which provided the basis for our independent pSS-ILD clinical validation.

The strong correlation between SPP1 and DLCO% predicted is another important finding. DLCO% predicted reflects alveolar-capillary integrity and is a sensitive indicator of gas exchange impairment, often declining before FVC. Iturbe-Fernández et al. (43) reported a negative correlation between serum SPP1 and FVC in 344 ILD patients. Gao et al. (44)further demonstrated that elevated serum SPP1 levels in systemic sclerosis-associated ILD (SSc-ILD) patients could predict future lung function deterioration. These findings suggest a consistent association between SPP1 and lung function decline across ILD subtypes. Notably, SPP1 correlated with DLCO% but not with FVC in our study. This finding may be explained by the distinct pathophysiology of pSS-ILD, where lymphocytic infiltration of the interstitium and peribronchiolar regions leads to early gas exchange impairment, whereas volume restriction develops later (45). Clinical studies have shown that DLCO impairment is common in pSS-ILD, whereas FVC may remain stable over time (46, 47). Therefore, the specific association between SPP1 and DLCO may reflect the disease evolution pattern in pSS-ILD, where diffusion impairment precedes restrictive ventilatory defects.

The elevation of serum SPP1 in pSS-ILD patients is consistent with previous reports in IPF and other CTD-ILD (23–25). And, Tsitoura et al. (23) found elevated SPP1 levels in bronchoalveolar lavage fluid from ILD patients, with airway macrophages co-expressing COL1A1 and SPP1, indicating an increase in profibrotic airway macrophages. These findings further support the potential of SPP1 as a common biomarker for ILD of diverse etiologies. Moreover, SPP1 is closely linked to AE of ILD. Previous studies have confirmed that serum SPP1 levels are significantly higher in AE-IPF patients than in stable patients, and positively correlate with CRP and LDH (48). Similarly, Qiu et al. (24) found elevated SPP1 in rapidly progressive ILD associated with anti-MDA5-positive dermatomyositis. Recent multi-ILD cohort studies further confirmed SPP1 elevation in both AE-IPF and rapidly progressive IIM-ILD (25). In our pSS-ILD cohort, serum SPP1 was significantly positively correlated with D-dimer and CRP, and showed a positively trend toward correlation with LDH, consistent with the acute-phase and tissue-injury response that accompanies AE. Our study extends these findings to pSS-ILD, further suggesting that SPP1 may have potential as a cross-subtype biomarker for AE. Multivariate Cox regression confirmed SPP1 as an independent predictor of 1-year mortality, consistent with previous reports (25, 48). Notably, the differences in HR values for SPP1 across studies may be attributable to variations in ILD subtypes, baseline disease severity, and follow-up duration. Nevertheless, the consistent finding that SPP1 serves as an independent prognostic factor provides robust evidence for its incorporation into the clinical risk assessment system for pSS-ILD.

Several limitations warrant consideration. This single-center retrospective study had a modest sample size (n=88), limiting statistical power and generalizability. Due to the small number of mortality events (only 14 events), the wide confidence interval of the mortality model indicates that the prognostic estimates remain imprecise. The absence of longitudinal serum samples precluded assessment of SPP1 dynamics. And, the transcriptomic dataset (GSE47460) comprised mixed ILD subtypes without stratification, restricting subtype-specific analysis, and mechanistic exploration of SPP1 in pSS-ILD was not performed.

Future multicenter prospective studies with larger sample sizes are needed to validate the prognostic value of SPP1 in pSS-ILD and to establish standardized detection protocols and reference thresholds. In addition, other candidate markers such as KIAA0125, FCRL5, and TNFRSF17 (sBCMA) were also significantly upregulated in the ILD transcriptomic dataset and may merit further investigation in pSS-ILD. And, combining SPP1 with other biomarkers such as KL-6 and SP-D may improve risk stratification via multi-marker models. Advanced technologies such as single-cell sequencing and spatial transcriptomics could clarify SPP1’s cellular sources and signaling pathways in pSS-ILD. This would inform its pathogenic mechanisms and ultimately enable interventional studies targeting SPP1 or its downstream pathways.

## 5 Conclusion

In this study, transcriptomic analysis revealed significant upregulation of SPP1 in ILD lung tissues. Clinical validation in our pSS-ILD cohort confirmed that serum SPP1 effectively reflects disease activity, predicts acute exacerbation, and is independently associated with 1-year mortality. These findings indicate that SPP1 is a promising non-invasive biomarker, warranting further validation in prospective studies.

## Data Availability

Publicly available datasets were analyzed in this study. This data can be found here: NCBI GEO, accession number GSE47460.
