# Supplementary material for: Serum SPP1 levels are associated with disease severity and 1-year mortality in primary Sjögren’s syndrome-associated interstitial lung disease
Source: Front Immunol. 2026 Oct 12;17:1959993. doi: 10.3389/fimmu.2026.1959993 (PMC13699488; doi:10.3389/fimmu.2026.1959993)
Supplement: Supplementary file 1 [file Supplementaryfile1.docx]

**Supplementary Table S1. Distribution of HRCT imaging patterns in pSS-ILD patients**

| **Imaging pattern** | **ALL (n=88)** | **SD (n=60)** | **AE (n=28)** | **P** |
| --- | --- | --- | --- | --- |
| **NSIP** | 62 (70.5%) | 44 (73.3%) | 18 (64.3%) | 0.386 |
| **UIP** | 21 (23.9%) | 12 (20.0%) | 9 (32.1%) | 0.213 |
| **LIP** | 4 (4.5%) | 3 (5.0%) | 1 (3.6%) | 0.764 |
| **OP** | 1 (1.1%) | 1 (1.7%) | 0 (0.0%) | 0.492 |

HRCT images were retrospectively analysed and independently examined by two experienced chest radiologists. Any disagreements between the two radiologists were resolved by consultation. Radiological findings were recorded as the non-specific interstitial pneumonia (NSIP) pattern, usual interstitial pneumonia (UIP) pattern, lymphocytic interstitial pneumonia (LIP) pattern, and organizing pneumonia (OP) pattern, based on the predominant pattern according to the 2013 American Thoracic Society classification of idiopathic interstitial pneumonia guidelines. P values were calculated using the chi-square test or Fisher's exact test, as appropriate. HRCT, high-resolution computed tomography; SD, stable disease; AE, acute exacerbation; pSS-ILD, primary Sjögren's syndrome-associated interstitial lung disease.

**Supplementary Table S2. Bootstrap internal validation of cut-offs and AUC values**

| **Outcome** | **Events / N** | **Apparent cut-off (ng/mL)** | **Bootstrap median cut-off (ng/mL)** | **Apparent AUC (95% CI)** | **Bootstrap-corrected AUC(95% CI)** |
| --- | --- | --- | --- | --- | --- |
| AE | 28 / 88 | 47.49 | 47.50 | 0.891 (0.827–0.955) | 0.891 (0.800–0.964) |
| Mortality | 14 / 88 | 40.41 | 40.40 | 0.712 (0.555–0.870) | 0.710 (0.537–0.860) |

Internal validation was performed using 1,000 bootstrap resamples with replacement. AE, acute exacerbation; AUC, area under the receiver operating characteristic curve; CI, confidence interval.

**Supplementary Table S3. Univariate Cox proportional hazard model for prognostic factors of pSS-ILD patients**

| **Univariate** | **p** | **q** | **HR** | **95%CI** |
| --- | --- | --- | --- | --- |
| Age, years | 0.032 | 0.100 | 1.065 | (1.005-1.127) |
| Sex, M(%) | 0.904 | 0.943 | 0.924 | (0.258-3.314) |
| Smoking history, n(%) | 0.742 | 0.851 | 0.778 | (0.174-3.475) |
| BMI, Kg/m^2^ | 0.473 | 0.642 | 0.931 | (0.766-1.132) |
| PO2, mmHg | 0.560 | 0.699 | 0.992 | (0.968-1.018) |
| PO_2_/FiO_2_, mmHg | <0.001 | 0.002 | 0.988 | (0.982-0.994) |
| D-dimer,mg/L | 0.039 | 0.115 | 1.057 | (1.003-1.114) |
| WBC, 10^9^/L | 0.010 | 0.047 | 1.215 | (1.047-1.410) |
| RBC, 10^12^/L | 0.446 | 0.633 | 0.670 | (0.240-1.874) |
| LYM%, % | 0.102 | 0.214 | 0.958 | (0.911-1.008) |
| LYM,10^9^/L | 0.143 | 0.271 | 0.512 | (0.021-1.253) |
| Hb, g/L | 0.810 | 0.886 | 0.996 | (0.963-1.030) |
| PLT, 10^9^/L | 0.060 | 0.152 | 0.993 | (0.987-1.000) |
| N%, % | 0.020 | 0.083 | 1.053 | (1.008-1.100) |
| N,10^9^/L | 0.002 | 0.013 | 1.239 | (1.080-1.415) |
| Alb, g/L | 0.369 | 0.581 | 0.943 | (0.829-1.072) |
| TCHO, mmol/L | 0.087 | 0.194 | 1.445 | (0.948-2.203) |
| TG, mmol/L | 0.108 | 0.216 | 1.804 | (0.879-3.703) |
| ALT, U/L | 0.026 | 0.089 | 1.014 | (1.002-1.026) |
| AST, U/L | 0.048 | 0.131 | 1.020 | (1.000-1.041) |
| CREA, umol/L | 0.072 | 0.173 | 1.024 | (0.998-1.051) |
| BUN, mmol/L | 0.007 | 0.041 | 1.162 | (1.041-1.297) |
| GLU, mmol/L | <0.001 | 0.001 | 1.562 | (1.307-1.867) |
| GLB, g/L | 0.446 | 0.633 | 1.026 | (0.960-1.096) |
| LDH, U/L | <0.001 | 0.002 | 1.004 | (1.002-1.005) |
| TP, g/L | 0.815 | 0.886 | 1.008 | (0.944-1.077) |
| CRP, mg/L | 0.002 | 0.013 | 1.023 | (1.009-1.038) |
| HDL, mmol/L | 0.170 | 0.308 | 1.429 | (0.858-2.378) |
| LDL, mmol/L | 0.725 | 0.851 | 0.902 | (0.509-1.600) |
| CK, U/L | 0.247 | 0.427 | 1.003 | (0.998-1.007) |
| CK-MB, U/L | 0.022 | 0.083 | 1.087 | (1.012-1.167) |
| C3, g/L | 0.513 | 0.672 | 0.414 | (0.029-5.832) |
| C4, g/L | 0.568 | 0.699 | 4.819 | (0.022-1066.866) |
| IgA, g/L | 0.275 | 0.452 | 1.204 | (0.863-1.679) |
| IgE, mg/L | 0.450 | 0.633 | 0.999 | (0.996-1.002) |
| IgG, g/L | 0.998 | 0.990 | 1.001 | (0.918-1.090) |
| IgM, g/L | 0.917 | 0.943 | 0.984 | (0.726-1.334) |
| SPP1, ng/mL | <0.001 | 0.003 | 1.017 | (1.008-1.027) |

Alb, albumin; ALT, alanine aminotransferase; AST, aspartate aminotransferase; BMI, body mass index; BUN, blood urea nitrogen; C3, complement 3; C4, complement 4; CK, creatine kinase; CK-MB, creatine kinase isoenzyme MB; CRP, C-reactive protein; CREA, creatinine; GLB, globulin; GLU, glucose; HDL, high-density lipoprotein; Hb, hemoglobin; HR, hazard ratio; IgA/G/M, immunoglobulin A/G/M; IgE, immunoglobulin E; LDH, lactate dehydrogenase; LDL, low-density lipoprotein; LYM, lymphocyte count; LYM%, lymphocyte percentage; N, neutrophil count; N%, neutrophil percentage; PLT, platelet count; PO2, partial pressure of oxygen; PO2/FiO2, ratio of partial pressure of oxygen to fraction of inspired oxygen; RBC, red blood cell count; SPP1, secreted phosphoprotein 1; TCHO, total cholesterol; TG, triglyceride; TP, total protein; WBC, white blood cell count; 95% CI, 95% confidence interval. The p column represents the unadjusted P-value from univariate Cox regression. The q column represents the FDR-adjusted P-value (q-value) using the Benjamini-Hochberg method to control for multiple comparisons. Variables with q < 0.05 were considered statistically significant.

**Supplementary Table S4. Sensitivity analyses of serum SPP1 as an independent prognostic factor for 1-year mortality in pSS-ILD patients**

| **Model** | **Additional covariate** | **SPP1 HR** | **95% CI (lower)** | **95% CI (upper)** | **P** |
| --- | --- | --- | --- | --- | --- |
| **Primary model** | None (age + sex only) | 1.016 | 1.006 | 1.025 | 0.001 |
| Sensitivity 1 | + PO_2_/FiO_2_ | 1.011 | 1.001 | 1.022 | 0.039 |
| Sensitivity 2 | + D-dimer | 1.014 | 1.004 | 1.025 | 0.007 |
| Sensitivity 3 | + BUN | 1.014 | 1.005 | 1.024 | 0.003 |
| Sensitivity 4 | + GLU | 1.013 | 1.000 | 1.026 | 0.020 |
| Sensitivity 5 | + CRP | 1.012 | 1.000 | 1.023 | 0.044 |
| **Firth-penalized** | None (age + sex only) | 1.015 | 1.006 | 1.025 | 0.002 |

A series of one-covariate-at-a-time Cox proportional hazards regression models were constructed. The primary model included SPP1, age, and sex; Each sensitivity model additionally included one baseline covariate. A Firth-penalized Cox regression was performed to correct for potential estimation bias due to the limited number of events (n=14). White blood cell count (WBC), neutrophil count (N), alanine aminotransferase (ALT), and lactate dehydrogenase (LDH) were excluded from the sensitivity models owing to collinearity with SPP1 (assessed by variance inflation factor). Across all models, the hazard ratio (HR) of SPP1 remained consistent in direction and magnitude (HR ≈ 1.01–1.02), indicating that its independent prognostic value for 1-year mortality was robust. HR, hazard ratio; CI, confidence interval; PO₂/FiO₂, ratio of arterial oxygen partial pressure to fractional inspired oxygen; BUN, blood urea nitrogen; GLU, glucose; CRP, C-reactive protein; pSS-ILD, primary Sjögren's syndrome-associated interstitial lung disease.

**Supplementary Figure S1. Co-expression network of SPP1 and functional enrichment of its co-expressed genes in ILD.** (A) Co-expression network showing SPP1 and its top 50 co-expressed genes in the GSE47460 dataset. The purple node represents SPP1, and green nodes represent co-expressed genes. Red edges indicate positive correlation, and blue edges indicate negative correlation. (B) Gene Ontology (GO) enrichment analysis of SPP1 co-expressed genes. The bubble plot displays significantly enriched biological processes, with the x-axis representing the gene ratio, the y-axis representing GO terms, node size indicating the number of genes, and node color indicating the adjusted P value (p.adjust). ILD, interstitial lung disease; SPP1, secreted phosphoprotein 1; GO, Gene Ontology.

**Supplementary Figure S2. Correlations of serum SPP1 with acute-phase and injury markers in pSS-ILD patients.** Scatter plots showing Spearman's rank correlations between serum SPP1 levels and PaO₂/FiO₂ (A), D-dimer (B), CRP (C), and LDH (D) in the overall cohort (n = 88). The pink line represents the fitted linear regression line with the 95% confidence band. Correlation coefficients (r), unadjusted P values, Benjamini-Hochberg false discovery rate (FDR)-corrected q values, and the number of paired observations (n) are shown in the inset of each panel. SPP1 was significantly and positively correlated with D-dimer (r = 0.378, P < 0.001, q = 0.005) and CRP (r = 0.282, P = 0.011, q = 0.039), whereas its correlations with PaO₂/FiO₂ (r = −0.195, P = 0.069, q = 0.125) and LDH (r = 0.236, P = 0.031, q = 0.085) did not remain statistically significant after FDR correction. SPP1, secreted phosphoprotein 1; pSS-ILD, primary Sjögren's syndrome-associated interstitial lung disease; PaO₂/FiO₂, ratio of arterial oxygen partial pressure to fractional inspired oxygen; CRP, C-reactive protein; LDH, lactate dehydrogenase; FDR, false discovery rate.
